# New Lymphogranuloma Venereum *Chlamydia trachomatis* Variant, Amsterdam

**DOI:** 10.3201/eid1107.040883

**Published:** 2005-07

**Authors:** Joke Spaargaren, Han S.A. Fennema, Servaas A. Morré, Henry J.C. de Vries, Roel A. Coutinho

**Affiliations:** * Municipal Health Service, Amsterdam, the Netherlands;; † VU University Medical Center, Amsterdam, the Netherlands;; ‡ Academic Medical Center, Amsterdam, the Netherlands;; § National Institute of Public Health and Environment, Bilthoven, the Netherlands

**Keywords:** Chlamydia trachomatis, genotype, LGV, omp A gene, L2 variant, L2b

## Abstract

We retrospectively conducted a study of men who have sex with men who visited the Amsterdam, the Netherlands, sexually transmitted diseases clinic from January 2002 to December 2003 and had rectal *Chlamydia trachomatis* infections. We found that symptomatic (73%) as well as asymptomatic (43%) patients were infected with a new *C. trachomatis* LGV variant.

In December 2003, an unusual symptom of early lymphogranuloma venereum (LGV) in a patient infected with HIV-1, who also had proctitis, was reported in Rotterdam, the Netherlands (*1*). In the same city, an outbreak of LGV with similar symptoms, such as proctitis and constipation, subsequently was identified in men who have sex with men (MSM) (*2*). Here we report 32 patients with, and 13 patients without, mucous membrane abnormalities in MSM with confirmed LGV in 2002–2003.

LGV is a systemic disease caused by the *Chlamydia trachomatis* serovars L1 to L3. More invasive than disease caused by the urogenital serovars (D–K), LGV can manifest as 1) an inguinal syndrome, with genital ulceration and inguinal lymphadenopathy (buboes) and subsequent suppuration, and 2) an anogenitorectal syndrome, with proctocolitis and hyperplasia of intestinal and perirectal lymphatic tissue. Both syndromes can be accompanied by systemic symptoms including fever, malaise, chills, anorexia, myalgia, and arthralgia. If left untreated, the infection can lead to fistulas, strictures, genital elephantiasis, frozen pelvis, and infertility (*3*). LGV is endemic in Africa, Southeast Asia, and the Caribbean; it is a sporadic disease in Europe and North America.

## The Study

For this study, we selected MSM who were treated at our sexually transmitted disease (STD) clinic (≈20,000 new consultations per year) in 2002 and 2003 with *C. trachomatis* proctitis confirmed by a positive polymerase chain reaction (PCR), COBAS AMPLICOR (Hoffman-La Roche Ltd., Basel, Switzerland). Upon proctoscopic examination by 1 medical practitioner, patients were designated into 2 groups: 1 group with mucous membrane abnormalities (MMA+, n = 44) when mucopurulent anal discharge or bloody, ulcerative rectal lesions were found, and 1 group without MMA (MMA–, n = 30) when those symptoms were not found. Samples were taken by proctoscopic examination. During the study, *C. trachomatis* proctitis was diagnosed in some patients at separate times. Those follow-up samples were excluded from the study. Calculations are based on the number of patients in whom *C. trachomatis* proctitis was diagnosed during their first visit. Patients were treated with a single dose of 1 g azithromycin, the consensus treatment for uncomplicated urogenital *C. trachomatis* infections at that time. Purified *C. trachomatis* DNA obtained from the rectal samples of these 74 patients was used to assess *C. trachomatis* serovars identified by PCR, based on restriction fragment length polymorphism (RFLP) analysis of the *omp*A gene as described previously (*4**,**5*). In addition, we sequenced the complete *omp*A gene to identify possible changes at the nucleotide level (ABI 310 automated sequencer, PE Biosystems, Nieuwerkerk a/d IJssel, the Netherlands). The exact sequence methods and primers are described by Morré et al. (*5*). In short, *omp*A nucleotide sequence analysis was performed in several sequence reactions generating the complete 1.1-kbp order. DNA sequencing was performed in both directions and analyzed by automated DNA sequencing on an ABI 310 sequencer. Sequences were aligned with the BioEdit Sequence Alignment Editor (Ibis Therapeutics, Carlsbad, CA, USA). Reference sequences were derived from GenBank (http://www.ncbi.nlm.nih.gov/GenBank).

Serum samples from these patients, taken at consultation and stored at –20°C, were used to measure *C. trachomatis*–specific immunoglobulin (Ig) G. This *C. trachomatis*–IgG peptide enzyme-linked immunosorbent assay (pELISA) (Medac Diagnostika mbH, Hamburg, Germany) is based on a synthetic peptide from an immunodominant region of the major outer membrane protein and was performed according to the manufacturer's instructions, as described previously (*6*). A titer of both ELISAs of ≥1:50 was considered positive, and an arbitrary differentiation was made between low (1:50–1:200) and high titers (>1:200).

Genotyping the *omp*A gene by RFLP of these 74 patients showed that 45 samples were positive for *C. trachomatis* all type L2 (Table). Sequencing of the *omp*A gene demonstrated that all L2-positive samples contained a new (based on the National Center for Biotechnology Information BLAST queries) *C. trachomatis* genovariant (Figure), which we designated L2b. The novel sequence was deposited in GenBank (accession no. AY586530).

**Table:** Characteristics of men who have sex with men in retrospective study at sexually transmitted disease outpatient clinic in Amsterdam, the Netherlands*

Clinical data	MMA+			MMA–		
	(2002 and 2003)			(2003)		
	*C. trachomatis*			*C. trachomatis*		
	L2b	Non-LGV	Total	L2b	Non-LGV	Total
No. patients	32	12†	44	13	17‡	30
Anal discharge	11	3	14	4	2	6
Genital ulcers	3 (L2b)	1 (*Treponema pallidum*)	4	0	1 (HSV-2)	1
Lymphadenopathy	4	0	4	2	2	4
Mean no. infections with sexually transmitted diseases	8.3	5.8	7.8	7	4.6	5.8
Known HIV+	24	6	30	7	7	14
>10 leukocytes by Gram stain	26	3	39	3	6	9
*C. trachomatis* lgG ≥200	22	2	24	8	6	14
*C. trachomatis* lgG <200	8§	10	18	4¶	11	15

*MMA, mucous membrane abnormalities; *C. trachomatis*, *Chlamydia trachomatis*; LGV, lymphogranuloma venereum; Ig, immunoglobulin. †2 serum samples were not available for testing. ‡1 serum sample was not available for testing. §Serogroup B, n = 5; intermediate serogroup, n = 5; serogroup C, n = 2. ¶Serogroup B, n = 10; intermediate serogroup, n = 1; serogroup C, n = 6.

**Figure:**
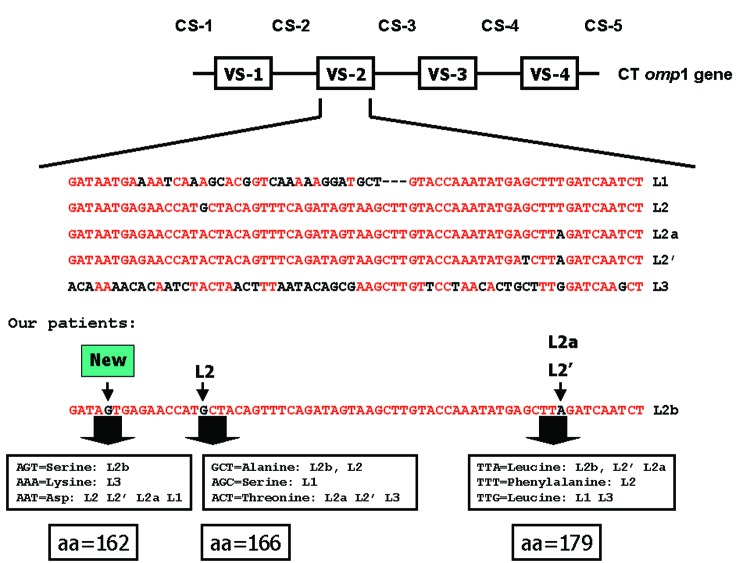
Schematic representation of the *Chlamydia trachomatis omp*A gene. In detail, variable segment 2 (VS-2): nucleotide and amino acid (aa) sequence comparison of the prototypes L1, L2, L2´, L2a, and L3 and the newly identified lymphogranuloma venereum (LVG) strain, which we designated L2b. Conserved nucleotides in VS-2 for all LGV strains are shown in red. The nucleotide substitutions in L2b as compared to all LGV strains is indicated by arrows. All aa encoded by the substitution combinations are indicated. CS = constant segment; omp = outer membrane protein.

When the *omp*A sequences of these patients were compared to the prototype sequences of L2 and its variants L2a and L2´, besides 2 already described changes, a new base pair change was found. One change in variable segment 2 was deducted from L2a and L2´, and one from L2. The third change has not been described before. All nucleotide changes resulted in amino acid substitutions. The fourth change was found in constant segment 2 (CS-2) at amino acid 157: the third nucleotide is G in L2b and L1, C in L2, and A in L3. As expected, this mutation is conserved, and all combinations encode for the amino acid glycine. Combining the sequence data with the RFLP typing showed that 32 of 44 samples from MMA+ and 13 of 30 samples from MMA– patients were L2b. In the MMA+ patient group, a positive chlamydia serologic test results mainly an IgG titer 1:≥200, correlated well with the LGV diagnosis. Approximately 80% of all LGV patients had high titers; in the MMA– group, species-specific *C. trachomatis* serologic test results did not correlate with LGV. The patients' characteristics are shown in the Table. Median age of the 45 men with samples positive for *C. trachomatis* was 35.8 years (range 25.9–47.6) compared with 38.1 years (range 25.8–58.2) for the men with samples negative for *C. trachomatis*. All *C. trachomatis*–positive patients lived in the Netherlands, most in Amsterdam, and most were of Dutch ethnic background.

Anal discharge was reported by 15 of 20 patients with LGV. Genital ulcers (all localized to the perianal area) and inguinal lymphadenopathy were found in only a few patients. Ulcers in the 2 patients infected with a non-LGV *C. trachomatis* strain were caused by herpes simplex virus 2 and *Treponema pallidum*. In the 3 ulcers found in the MMA+ patients, the L2b *C. trachomatis* strain was found.

The mean number of previously documented sexually transmitted infection episodes was 8.3 among the MMA+ LGV patients in contrast to 5.8 episodes in the non-LGV patients. Twenty-four of 30 of the MMA+ LGV patients and 7 of 14 of the MMA– LGV patients were HIV-infected. All patients with a retrospective diagnosis of LGV were contacted and offered reexamination. If the L2b strain persisted, the patients received doxycycline, 100 mg twice daily for 3 weeks, the consensus treatment for LGV.

## Conclusions

We conclude the following: 1) the outbreak of LGV among MSM in the Netherlands expands beyond the cluster reported earlier in Rotterdam and can be traced back to at least January 2002; 2) the outbreak in Amsterdam and possibly the one in Rotterdam was caused by a newly identified L2b variant; 3) both MMA+ and MMA– men are infected with *C. trachomatis* and most of them are HIV-positive; 4) species-specific serology can help support the LGV diagnosis when clinical symptoms are present but cannot be used to detect LGV-infected persons who are asymptomatic.

Although based upon a small, select population, our results justify additional study of high-risk core groups who transmit this LGV genovariant to determine transmission risk factors and diagnostic criteria. The outbreak of LGV is ongoing; we currently see 1–2 new patients per week at our STD clinic.
